# Neonatal lupus with left bundle branch block and cardiomyopathy: a case report

**DOI:** 10.1186/s12872-020-01637-4

**Published:** 2020-07-29

**Authors:** Brad Rumancik, Anita N. Haggstrom, Eric S. Ebenroth

**Affiliations:** 1 Indiana University School of Medicine, Indianapolis, IN 46202 USA; 2 Department of Dermatology, Indiana University School of Medicine, Indianapolis, IN 46202 USA; 3 Department of Pediatrics, Section of Cardiology, Indiana University School of Medicine, 705 Riley Hospital Drive, Suite 1134, Indianapolis, IN 46202 USA

**Keywords:** Case report, Neonatal lupus, Dilated cardiomyopathy, Left bundle branch block, Anti-Sjögren’s-syndrome type a/Ro (anti-SSA/Ro), Anti-Sjögren’s-syndrome type B/La (anti-SSB/La), Intravenous immunoglobulin

## Abstract

**Background:**

Cardiac manifestations of neonatal lupus include an array of structural and conduction abnormalities due to placental transference of maternal anti-SSA/Ro and anti-SSB/La autoantibodies. Late-onset neonatal lupus cardiomyopathies, occurring outside the neonatal period, is an infrequently reported manifestation with unknown pathophysiology and poorly defined treatment regimens. Due to the rarity of this condition, additional studies and case reports are required to better understand and manage late-onset neonatal lupus cardiomyopathies.

**Case presentation:**

A 4-week-old female, born to a mother with known anti-SSA/Ro and anti-SSB/La autoantibodies, presents with classic cutaneous manifestations for neonatal lupus and is found to have left bundle branch block, severely dilated cardiomyopathy with an ejection fraction of 25%, and a thin echogenic dyskinetic ventricular septum. Weekly second trimester and 30-week fetal echocardiograms showed no signs of structural or conduction abnormalities. There were no histologic signs of inflammation on cardiac tissue biopsy. After a complicated hospital course, she was successfully treated with biventricular pacemaker, intravenous immunoglobulin, and plasmapheresis.

**Conclusions:**

We present a case of late-onset neonatal lupus with severe dilated cardiomyopathy, a dyskinetic ventricular septum, and left bundle branch block. To our knowledge, the dyskinetic ventricular septum has never been reported and left bundle branch block is rarely reported in NL. This case further validates the need for long term cardiac follow up for patients born with NL, even if lacking cardiac manifestations in the peripartum period. We characterize a unique presentation of a rare clinical entity, highlighting the diagnostic challenges, and describe a successful treatment course.

## Background

Neonatal lupus (NL) is caused by placental transfer of maternal anti-Sjögren’s-syndrome type A/Ro (anti-SSA/Ro) and anti-Sjögren’s-syndrome type B/La (anti-SSB/La) autoantibodies. The incidence of NL in newborns who have mothers with positive anti-SSA/Ro and anti-SSB/La autoantibody titers is approximately 1–2% [1–3]. Cutaneous and cardiac disease are the predominant manifestations [1, 4]. Conduction defects are the most common cardiac manifestation in NL with atrioventricular block (AVB) constituting the principle diagnosis [5]. Approximately 20% of cases develop diffuse cardiac disease most often manifesting as systolic dysfunction, ventricular dilation, and endocardial fibroelastosis (EFE) [6, 7]. Cardiac manifestations of NL typically occur in utero or within the neonatal period. However, delayed onset dilated cardiomyopathy beyond the neonatal period rarely has been reported [8–12]. Hypotheses suggest this rare presentation of NL-cardiomyopathy, defined as “late-onset,” results from a different mechanism compared to the cardiomyopathies occurring in utero or during the neonatal period [8, 9]. Management of NL-related cardiomyopathies has been challenging, often leading to heart transplant or poor outcomes despite pacemaker placement [6, 7, 11, 13]. Intravenous immunoglobulin (IVIG), corticosteroids, and plasmapheresis have been attempted with varying degrees of success for NL-related cardiomyopathies, but selection of therapy remains difficult due to the small number of reported cases [6, 12, 14]. We present a unique case of NL with severe dilated cardiomyopathy, a dyskinetic ventricular septum, and left bundle branch block (LBBB). Our case has conduction manifestations rarely reported, LBBB, and structural abnormalities never reported, dyskinetic ventricular septum. Lastly, we describe successful treatment with biventricular pacemaker, IVIG, and plasmapheresis.

## Case presentation

**Fig. 1:**
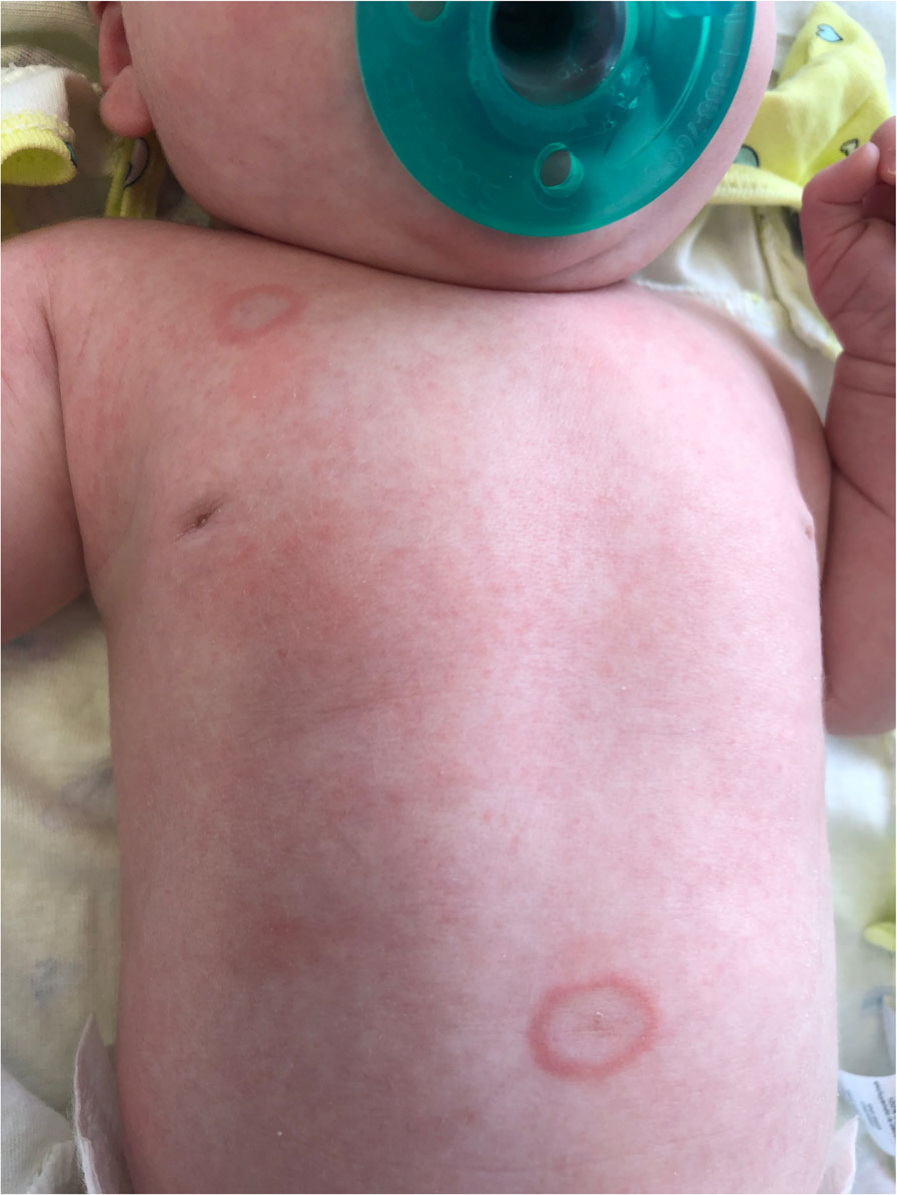
Erythematous annular lesions on the chest and abdomen of the patient, typical of neonatal lupus dermatitis

A 4-week-old female presented to the dermatology clinic with a 2-week history of erythematous annular lesions on the chest, abdomen, lower back, and bilateral posterior auricular regions consistent with NL (Fig. 1).

The 31-year-old mother, who is gravida 2, para 2, abortus 0 after delivery of the case-patient, has a history of systemic lupus erythematosus (SLE) and Sjögren syndrome with known anti-SSA/Ro and anti-SSB/La autoantibodies. She was diagnosed with SLE 7 years ago at 18 weeks gestation during her first pregnancy with her son. Her son, born at term, did not develop NL and is currently healthy with mild asthma. She has not seen a rheumatologist since her first pregnancy and takes no medications for SLE. There is no known or documented major organ involvement. She did not receive prenatal rheumatologic care or medications for SLE during her pregnancy with the case-patient. The mother retrospectively reported a self-limiting flare of arthritis in the last 2 weeks of the pregnancy. Maternal anti-SSA/Ro and anti-SSB/La autoantibody levels were not obtained during her second pregnancy. Weekly second trimester and 30-week fetal echocardiograms showed no structural abnormalities or bradycardia. There was no evidence of fetal 3rd degree AVB and the ventricular function was normal. The patient was born via an uncomplicated vaginal delivery at term weighing 3450 g with APGAR scores of 8 and 9 at 1 and 5 min, respectively. The newborn and mother were discharged to home after 24 h without any concern for NL or other medical conditions.

**Fig. 2:**
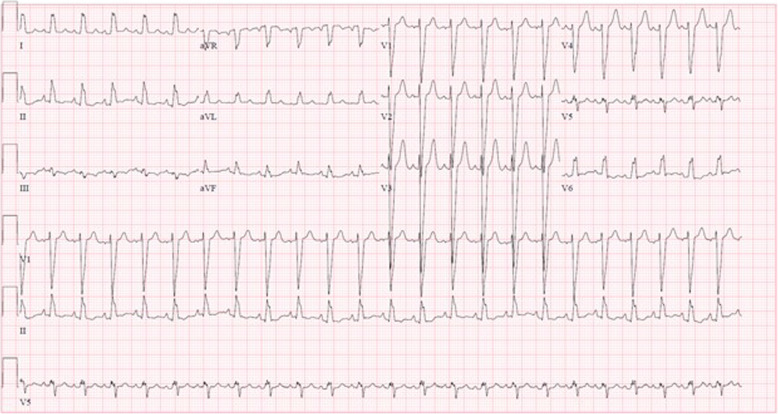
Electrocardiogram at 4-weeks of age showing left bundle branch block

**Fig. 3:**
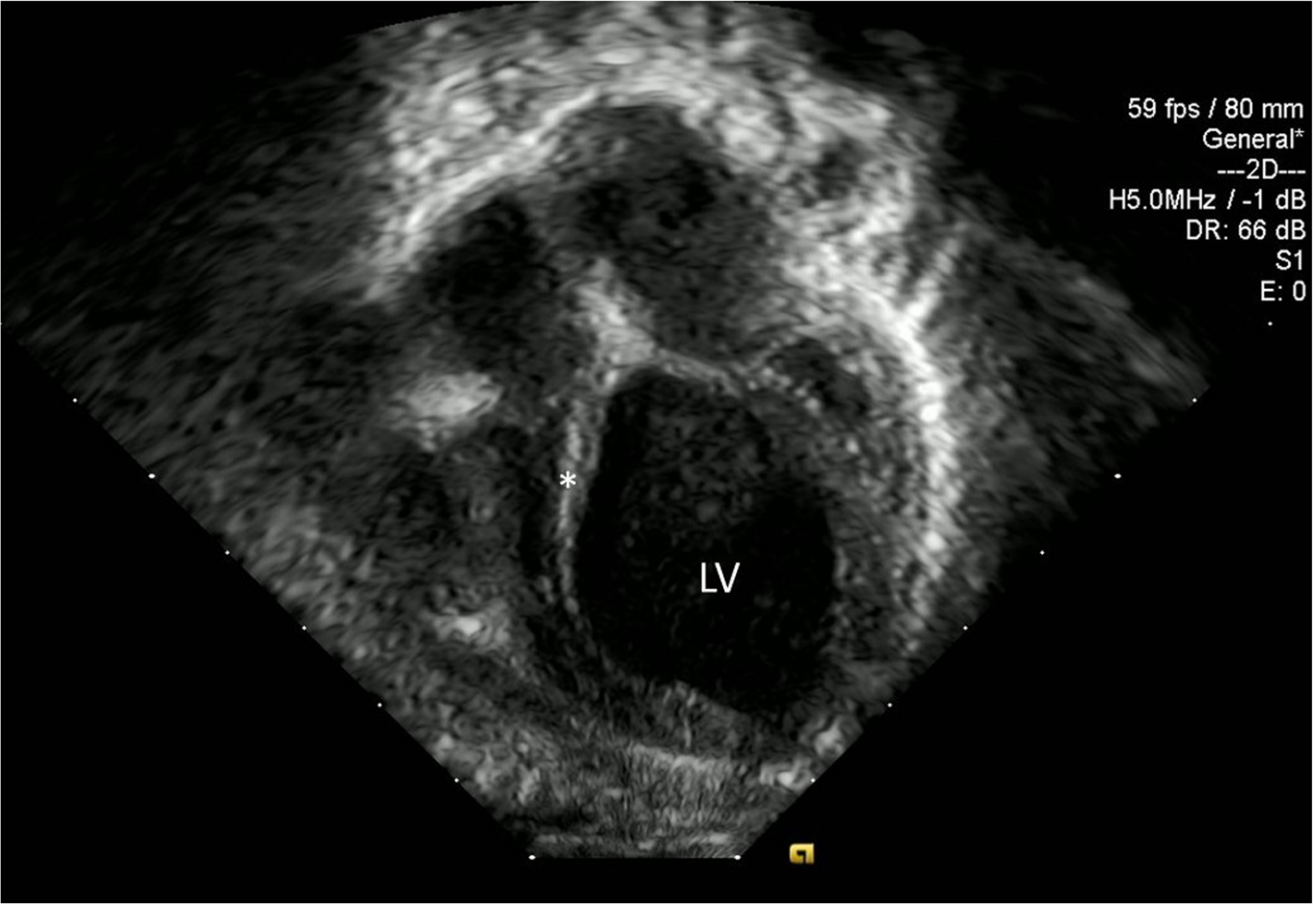
Echocardiography at 5 weeks of life showing severely dilated left ventricle (LV) and thin echogenic, dyskinetic ventricular septum (*). The origins of coronary arteries were normal with an ejection fraction of 25%, mild tricuspid insufficiency, and mild mitral insufficiency

The patient initially did well with appropriate weight gain and no cyanosis, shortness of breath, or diaphoresis when feeding. The cutaneous findings, starting at 2 weeks of life, led to a screening electrocardiogram at 4-weeks of life which showed LBBB (Fig. 2). Echocardiography at 5 weeks of life revealed severely dilated cardiomyopathy with an ejection fraction of 25%, a thin echogenic dyskinetic ventricular septum, normal appearing origins of coronary arteries, mild tricuspid insufficiency, and mild mitral insufficiency (Fig. 3). Despite adequate weight gain and no symptoms other than the rash, the patient was admitted for further workup and medical management of her heart failure.

Initial blood testing confirmed the diagnosis of NL by revealing an antinuclear antibody titer of 1:640 (normal value: less than 1:80) and robust anti-SSA/Ro and anti-SSB/La autoantibody levels greater than 8 enzyme-linked immunosorbent assay (ELISA) units (normal value: less than 1 ELISA unit). Screening for lysosomal acid alpha-glucosidase deficiency (Pompe disease), muscular dystrophies, Barth syndrome, fatty acid oxidation disorders, mitochondrial disorders, congenital disorders of glycosylation, mucopolysaccharidoses, and a cardiomyopathy genetic screening panel (a molecular evaluation for hereditary causes of cardiomyopathy; screening for abnormalities in over 100 known genes) were all negative. Cardiac enzymes and screening for viral pathogens was not obtained. She was started on heart failure management consisting of aspirin, captopril, furosemide, and carvedilol. LBBB persisted but no AVB was detected during the hospital stay. The patient was discharged on day 5 with stable hemodynamics and a plan to further titrate the heart failure medications as tolerated.

Two-month and 3-month follow up echocardiography were unchanged from her first study. She was admitted at 3-months of age for intermittent tachypnea and diaphoresis with feeding. Frequent non-sustained atrial tachycardia was discovered, and she was started on digoxin. Due to her persistent myopathy, and the unusual appearance of her thin, echobright, dyskinetic ventricular septum, she was taken to the cardiac catheterization laboratory to assess for other possible causes of perinatal infarction. Cardiac catheterization revealed unremarkable, patent coronary arteries. Endomyocardial biopsy of the right ventricle obtained during catheterization revealed no significant histologic abnormalities or antibody deposition. During catheterization, the patient developed bradycardia which was non-responsive to epinephrine and led to pulseless electrical activity requiring 44 min of emergent resuscitation and extracorporeal membrane oxygenation (ECMO). During the subsequent cardiovascular intensive care unit stay, repeat anti-SSA/Ro and anti-SSB/La autoantibodies at 3 months of life were 4.7 ELISA units and greater than 8 ELISA units, respectively. A biventricular pacemaker was placed at 3 months and 12 days of life which converted the LBBB to a narrow complex paced rhythm with significant improvement in septal dyskinesia and stroke volume. Plasmapheresis at 3 months and 13 days of life resulted in a decrease of anti-SSA/Ro and anti-SSB/La autoantibody from 2.4 ELISA units and 5.2 ELISA units to 0.1 ELISA units and 1.3 ELISA units, respectively. At 3 months and 14 days of life one dose of IVIG 1 g/kg was given, and the patient was successfully weaned off ECMO after 7 days of therapy (lasting from 3 months and 8 days to 3 months and 14 days of life). Subsequent echocardiography 6 days after stopping ECMO revealed improved systolic function with an ejection fraction of 30%. She was medically optimized and discharged on captopril, digoxin, carvedilol, furosemide, and aspirin.

At 8 months of age, approximately 4 months after discharge, she had a narrower LBBB (QRS duration 100 msec) paced rhythm with her biventricular pacemaker working appropriately. Echocardiography revealed normal left ventricular volume and systolic function (ejection fraction 70%), and furosemide and aspirin were discontinued. She has remaining neurologic complications from the emergent resuscitation; however, she is starting to achieve milestones and has reestablished an appropriate weight and height percentile for her age.

## Discussion and conclusions

Cardiac structural abnormalities, such as dilated cardiomyopathy and valvular disorders, are well-reported sequelae of NL, yet this patient’s thin, dyskinetic ventricular septum is a structural abnormality which has not been reported in association with NL [13, 15]. Coronary catheterization was performed to assess for evidence of perinatal infarction of the septum, possibly related to antibody-mediated coronary artery inflammation. The coronary catheterization revealed no abnormalities, and the endomyocardial biopsy showed no signs of inflammation, antibody reaction, or infarction. The etiology of the dyskinetic septum is not well-defined; however, it is likely similar to the underlying pathology leading to the patient’s dilated cardiomyopathy. Endomyocardial biopsies in NL-related dilated cardiomyopathy are often associated with immunoglobulin deposition and other inflammatory signs [4, 13]. However, there are reports of anti-SSA/Ro and/or anti-SSB/La positive patients with dilated cardiomyopathy who are found to have biopsies negative for inflammation or immunoglobulin deposition [8, 10–12]. Morel et al. proposed a distinct subtype of NL-related postnatal dilated cardiomyopathy [8]. This subtype is defined as “late-onset” and lacks histologic evidence of inflammation or immune deposits. Morel et al. suggested the later onset of clinically significant cardiomyopathy is precipitated by an unknown postnatal factor, possibly related to a virus or pacemaker placement (in those patients with AVB). Estimates for median age of onset of late-onset dilated cardiomyopathy include 11.5 months (range: 14 days to 9.3 years) and 15.2 months (range: 3.6 months to 22.8 years) [8, 11, 12]. In these reported cases, spontaneous development of dilated cardiomyopathy with marked reduction in ejection fractions occurred in patients who previously had either mild clinical findings of NL (characteristic of our patient) or required perinatal pacemaker placement. Given our patient’s benign fetal echocardiography, her structural defects associated with negative histologic findings may be related to this same “late-onset” process. Lastly, the reported timeline for median age of onset of late-onset dilated cardiomyopathy warrants long term follow up in patients born with NL, even if lacking cardiac manifestations in the peripartum period.

The lack of histologic immunoglobulin deposition, which has been reported in late-onset dilated cardiomyopathy, creates a challenge to definitively explain an etiology. The most common cause of pediatric dilated cardiomyopathy is idiopathic (approximately 66% of the cases), while frequently identified causes are related to myocarditis, neuromuscular disorders, familial cardiomyopathies, errors in metabolism, and congenital malformations [16]. Neuromuscular disorders, such as muscular dystrophies, and errors in metabolism, such as Pompe disease, were ruled out. The patient has no known family history of heart disease so familial cardiomyopathy is not likely. There were no viral prodromal symptoms prior to presentation and endomyocardial biopsy was negative for any signs of infective myocarditis. Although we cannot completely rule out the possibility of a coincidental idiopathic dilated cardiomyopathy, based on the extensive negative workup, and the presence of other NL symptoms, we do not suspect this to be the case. Our patient had classic NL cutaneous findings, high anti-SSA/Ro and anti-SSB/La titers, electroconductive pathology, and cardiomyopathy – all unifying sequelae well-described in NL. The mechanism of late-onset NL is not yet elucidated, but anti-SSA/Ro and anti-SSB/La titers are believed to be predictive factors for developing late-onset dilated cardiomyopathy [8–10]. The annual incidence of pediatric cardiomyopathies in the United States is 1.1–1.5 cases per 100,000, and the incidence of pediatric dilated cardiomyopathy is 0.56 cases per 100,000 person years [17, 18]. In contrast, the incidence for late-onset dilated cardiomyopathy in patients with positive anti-SSA/Ro and anti-SSB/La titers ranges from 5 to 14.9% [8–10, 19]. Lastly, our patient shares characteristics of other published cases of late-onset dilated cardiomyopathy in NL with and without histologic evidence of myocardial inflammation [8, 10–12].

The classic cardiac manifestation in NL is AVB which most often occurs with a structurally normal heart [4]. Other arrhythmias and conduction abnormalities have been reported including sinus node dysfunction, QT prolongation, and low QRS voltage [5, 13]. Bundle branch pathology in NL has been infrequently reported, and we are aware of only 3 other cases of LBBB as described by Wang et al. [5, 14, 20]. Of note, the structural pathology of our patient’s septum could explain or contribute to the LBBB independent of any antibody-mediated pathology.

It is rare to see structural heart disease in NL in the absence of AVB, and the incidence of any structural heart disease is unknown in anti-SSA/Ro and/or anti-SSB/La positive patients without AVB [4, 13, 21]. Nield et al. reported the first 3 cases of isolated EFE associated with anti-SSA/Ro and anti-SSB/La antibodies in the absence of AVB. The authors suggested a separate pathophysiologic cardiac manifestation of NL apart from the process which causes the classic AVB. Immunoglobulin (Ig) M, IgA, and the occasional B-cell was found in the cardiac tissue suggesting a secondary ongoing postnatal autoimmune reaction following the initial maternal antibody-mediated myocardial damage in utero. This suggested postnatal process may lead to a variety of structural changes including EFE and ventricular dilation [7]. However, our case is unique in that the late onset structural heart disease occurred in the absence of histologic evidence of inflammation.

The hypothesis of an ongoing postnatal autoimmune reaction led to the further investigation into a variety of prenatal and postnatal IVIG and corticosteroid regimens in patients with autoantibody-mediated fetal cardiomyopathies [6]. Trucco et al. demonstrated that IVIG and corticosteroid treatments showed some promise as 16 of 20 (80%) patients were alive with normal ventricular function at median follow up of 2.9 years. EFE was the identified structural abnormality in 16 patients and the remaining 4 patients had only systolic dysfunction. Of note, EFE was diagnosed with echocardiography and there were no confirmatory histologic samples [6]. The aforementioned Wang et al. manuscript describes 3 cases of NL with LBBB and dilated cardiomyopathy resembling our patient. There was no further description of cardiac tissue histologic analysis of the 3 patients. The 3 patients were treated with IVIG and glucocorticoids, did not receive a pacemaker, and are in stable condition now [14]. Despite the lack of histologic evidence for inflammation in our patient, IVIG and plasmapheresis were utilized. There was some concern for early development of EFE so plasmapheresis plus IVIG was thought to possibly mitigate any developing secondary immune process. However, the concern for EFE was largely clinical, lacking objective evidence, and IVIG and plasmapheresis was initiated partly due to the patient’s critical status. Pacemaker placement, plasmapheresis, then IVIG were administered in sequential days leading to improvement in ejection fraction in 6 days. It is impossible to say if IVIG and plasmapheresis provided any stabilization or if patient’s clinical status would have improved with pacemaker placement alone.

In summary, cardiac structural abnormalities may present postnatally in NL with or without histologic evidence of inflammation. The cardiac abnormalities may be delayed beyond the neonatal period necessitating the need for long term cardiac follow up in NL patients. The mechanism behind these structural abnormalities is still unclear. We present a NL case with rare structural and conduction abnormalities and negative histologic findings. The patient was successfully treated with biventricular pacemaker, IVIG, and plasmapheresis.

## Data Availability

Data and materials can be obtained for review by contacting the Corresponding Author.

## Abbreviations

NL: Neonatal lupus
LBBB: Left bundle branch block
AVB: 3rd degree atrioventricular block
ELISA: Enzyme-linked immunosorbent assay
ECMO: Extracorporeal membrane oxygenation
IVIG: Intravenous immunoglobulin
EFE: Endocardial fibroelastosis
